# Foeto–Maternal outcomes of pregnancies beyond 41 weeks of gestation after induced or spontaneous labour

**DOI:** 10.1016/j.eurox.2024.100339

**Published:** 2024-09-02

**Authors:** Sahruh Turkmen, Linnea Binfare

**Affiliations:** a Department of Clinical Sciences, Obstetrics and Gynecology, Sundsvalls Research Unit, Umeå University, Umeå, SE 90185, Sweden; b Department of Obstetrics and Gynecology, Sundsvall County Hospital, Sundsvall, SE 85186, Sweden

**Keywords:** Prolonged pregnancy, Induction of labour, Spontaneous onset of labour, Morbidity, Stillbirth, Caesarean section

## Abstract

**Objective:**

It has been suggested that induction of labour before 42 weeks of pregnancy prevents foetal complications. To evaluate the maternal and foetal outcomes of induced and spontaneous labour beyond gestational week 41 + 0.

**Study design:**

We conducted a register-based nationwide cohort study that included pregnant women who were delivered in Sweden in 2016–2021. Women were classified into two groups: induction of labour (IOL) or spontaneous onset of labour (SOL). Maternal and foetal outcomes after IOL in gestational week 41 were compared with SOL in gestational week 41 and 42.

**Results:**

Comparison between the IOL (n = 23,772) and SOL (n = 62,611) groups in gestational weeks 41 showed that various parameters were higher in the IOL group: caesarean deliveries (12.3 % and 4.6 %, *P* < 0.001), vacuum extraction (8.7 % and 6.9 %, *P* < 0.001), blood loss of > 1000 ml during labour (11 % vs 8.3 %, *P* < 0.001). The risks were remained significant even after adjusting for potential confounders (caesarean delivery: aOR 2.36; 95 % CI, 2.23–2.50, vacuum delivery: aOR 1.09; 95 % CI, 1.03–1.16, *P* = 0.002, and blood loss of >1000 ml: aOR 1.25; 95 % CI 1.18–1.31). The proportions of stillbirths (0.07 % and 0.18, *P* < 0.001), and newborns with apgar scores < 4 at five minutes (0.4 % vs 0.3 %, *P* < 0.001), were also higher in the IOL group. The risk of stillbirth after IOL in gestational week 41 was increased relative to SOL in the same week and remained high after adjusting for potential confounders (aOR 1.75; 95 % CI 1.07–2.80, *P* = 0.025).

The IOL group in gestational weeks 41 comprised a higher proportion of caesarean deliveries (12.3 % and 8.5 %, *P* < 0.001), but a lower (8.7 % and 9.7 %, *P* = 0.006) proportion of deliveries by vacuum extraction than the SOL group (n = 4548) in week 42.

**Conclusions:**

Inducing labour at gestational week 41 in women with prolonged pregnancies may have adverse effects on foetal and maternal outcomes compared to those who experience spontaneous labour onset at the same gestational age. The risk of negative foetal outcomes after induction at week 41 appears similar to that in women who give birth after spontaneous labour at week 42.

## Highlights

• IOL at 41 weeks may negatively impact foetal-maternal outcomes compared to SOL.
• Deliveries after SOL at 42 weeks may have similar foetal risks as IOL at 41 weeks.
• In late-term pregnancy, both fetal and maternal risks increase as gestational age advances.

## 1 Introduction

Prolonged pregnancy refers to gestation that extends 2 weeks or more beyond the estimated due date, and the term is used interchangeably with “post-date pregnancy” [1], [2]. Managing pregnancy in patients with late-term gestation is a challenge for clinicians. The current management of prolonged pregnancy has been questioned in several studies because emerging evidence indicates that the incidence of complications associated with prolonged pregnancy increases after the due date, even before 42 weeks of pregnancy. Pregnancy that extends beyond the due date is associated with an increased risk of intra-uterine foetal mortality and morbidity and increased maternal morbidity [3], [4], [5]. Several studies have suggested that the induction of labour before 42 weeks of pregnancy prevents these complications. However, there are concerns about the risks associated with induced labour, including failed induction, increased caesarean section rates, increased costs, and increased anxiety for the pregnant woman [6], [7], [8].

Earlier studies based on recorded data have concluded that maternal and foetal risks increase after 41 weeks, so closer monitoring should commence, and the induction of labour should be considered in gestational week 41 [9], [10], [11], [12]. A multi-centre study from Sweden (SWEPIS study) compared the induction of labour at 41 weeks with expectant management or induction at 42 weeks [13]. That study showed less perinatal mortality among pregnant women who commenced labour earlier (at 41 weeks), but no significant differences in the proportions of caesarean deliveries, instrumental vaginal deliveries, any major maternal morbidity, or perinatal outcomes between the strategies. It was subsequently recommended that the induction of labour should be offered to women within week 41 as a possible intervention to reduce the proportion of stillbirths. In recent years, clinical procedures for prolonged pregnancy have changed in Sweden, with earlier induction preferred. However, because induction is an intervention that can potentially harm both mother and child, it is important to ensure that the benefits of a change in clinical practice outweigh the harms.

In this study, we examined the maternal and foetal outcomes in women with prolonged low-risk pregnancies to determine the influence of the method of labour commencement (spontaneous or induced) on outcome parameters in different gestational ages.

## 2 Materials and methods

We conducted a register-based nationwide cohort study of pregnant women who gave birth in Sweden between 1 January 2016 and 31 December 2021. In Sweden, there are national guidelines for the care of pregnant women, childbirth and the postpartum period, which aim to ensure uniform and high-quality care throughout the country. Following a recommendation from the SWEPIS study results [13], several regions in Sweden have changed their routines for handling late term pregnancies and have started offering induction of labour to pregnant women as early as 41 weeks of pregnancy. Data for women who gave birth at a gestational age of 41 + 0 to 42 + 6 weeks were extracted from the national Swedish Pregnancy Register, which was established in 2013 and currently includes data on ∼90 % of pregnancies in Sweden. The Swedish Pregnancy Register includes detailed information on women’s pregnancies and deliveries, which are registered in their electronic medical records by midwives and doctors in a standardized manner at the first and each subsequent visit, ultrasound examination, delivery, and care visits [14]. The Ethics Review Authority of Stockholm Division 1 Medicine approved the study (Dnr 2020–06274).

**Fig. 1:**
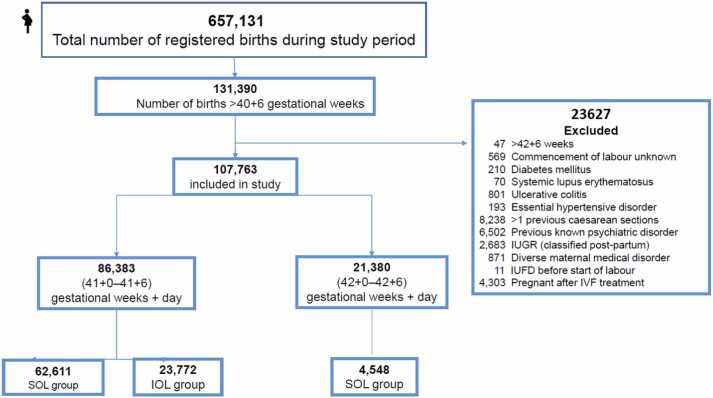
Flow chart of patient selection. SOL, spontaneous onset of labour (SOL); IOL, induction of labour (IOL).

The inclusion criteria were as follows: pregnant women aged ≥ 18 years and a spontaneous pregnancy involving one foetus with a cephalic presentation, whose gestational age exceeded 40 weeks and 6 days according to ultrasound-based dating in the first or early second trimester. Given the potential effects of various conditions on the foetus and the pregnant women, the exclusion criteria were as follows: more than one previous caesarean section (according to clinical practice in Sweden, patients with a history of one uncomplicated caesarean section are allowed to choose vaginal delivery) or other uterine surgery that resulted in an inability to deliver vaginally; reproductive-technology-assisted pregnancies; diabetes mellitus or gestational diabetes; hypertensive gestational disorder; oligohydramnios (amniotic fluid index < 50 mm or deepest vertical pocket < 20 mm); small for gestational age foetus (estimated foetal weight lower than the mean by ≥2 standard deviations); major congenital malformation causing significant functional impairment or life-limiting outcome (e.g., neural tube defects, heart defects); contraindication for vaginal delivery; and any other maternal disease that might affect the progress of pregnancy to gestational week 42 + 6 (see Fig. 1, flow chart). In this study, we aimed to evaluate the effect of labour commencement methods on foetal and maternal outcomes, therefore all IUFD before the start of labour were excluded. After the exclusion criteria were applied, to identify and exclude patients who experienced intra-uterine foetal death (IUFD) before the start of labour, data for patients who experienced stillbirth were matched with the neonatal diagnoses, and only stillbirths that occurred after the start of labour were included (Fig. 1).

Eligible women were divided into two groups according to delivery at gestational week 41 + 0–41 + 6 or 42 + 0–42 + 6. They were subsequently classified based on the method of labour commencement: spontaneous onset of labour (SOL) or induction of labour (IOL). We compared maternal and fetal outcomes between IOL at gestational week 41 + 0–41 + 6 and SOL at the same gestational age, as well as SOL at gestational week 42 + 0–42 + 6. Our study focused on the influence of the commencement method on maternal and fetal outcomes during these specific gestational weeks.

We used the women’s unique personal identification number to retrieve data on antenatal, delivery, and neonatal characteristics from the Swedish Pregnancy Register. The study parameters were divided into maternal and foetal outcomes. The primary maternal outcomes were the delivery method (caesarean section, vacuum extraction/non-instrumental vaginal delivery), perineal injury (grade 3 or 4), and proportion of patients with blood loss of > 1000 ml. The secondary maternal outcomes were age at delivery (years), Body Mass Index (BMI kg/m2), parity, previous caesarean delivery, and tobacco use.

The primary foetal outcomes were neonatal stillbirth (defined as intrapartum foetal death), and apgar score < 4 at 5 min (a score of 0–3 at 5 min or later defined as a non-specific sign of illness [15]. The secondary foetal outcomes were diagnosis of meconium aspiration, admission of newborns to the neonatal intensive care, and blood gases (pH, and base excess [BE] values) in the umbilical artery at birth.

### 2.1 Statistical analysis

All statistical analyses were performed using the Statistical Package for Social Sciences version 29 (SPSS - IBM Corp., Armonk, NY, USA). The primary statistical analysis was a comparison of the outcome parameters in the IOL and SOL groups, separately at gestational ages of 41 or 42 weeks, with Pearson’s χ^2^ test for dichotomous variables and Student’s *t-*test for continuous variables. The significance level was set to *P* < 0.05. To estimate the probability of an event occurring in the groups, we used a logistic regression analysis and calculated the crude odds ratio (cOR) and its 95 % confidence interval (CI). In case of significance, analyses were continued with the calculation of the adjusted odds ratio (aOR) by adjusting for confounding factors: age, body mass index (BMI), parity, birth weight, tobacco use during pregnancy, and previous caesarean delivery. The data are presented as the mean ± standard deviation (SD) for continue data and as percentages for dichotomous data.

The data was incomplete for some variables (missing at random), with the highest percentages of missing data observed for BE and PH (33.5 % and 29.7 %, respectively). Other parameters with missing data included BMI (6.2 %) and total bleeding > 1 liter (0.3 %). We conducted sensitivity analyses to address the missing data using multiple imputation with five repetitions. Statistical analyses were then performed on the pooled data set after imputation.

In the study by Wennerholm and colleagues [13], foetal mortality in the expectant management protocol group was reported to be 0.4 %, but 0.0 % in the group treated with an induction protocol. In our study, to identify the same difference in foetal mortality with α = 0.05 % and 80 % power, we required 1957 patients in each group.

## 3 Results

In total, 657,131 births were registered in the Swedish Pregnancy Register during the study period. After the inclusion and exclusion criteria were applied, 107,763 patients were ultimately included in the study. A total of 88,383 women delivered after spontaneous onset of labour (SOL) and induction of labour in gestational week 41 + 0–41 + 6. The number patients delivered after SOL and IOL in gestational 42 + 0–42 + 6 week was 21,380. (Fig. 1).

To determine the influence of the method of labour commencement (spontaneous or induced) on the outcome parameters in prolonged pregnancies, the maternal and foetal outcomes after IOL in gestational week 41 + 0–41 + 6 were compared with SOL in both gestational weeks 41 + 0–41 + 6 and 42 + 0–42 + 6.

To address the missing data, sensitivity analyses were performed using multiple imputation tests. The results showed that the percentage changes in outputs were marginal compared to the original complete-case data; however, the significance of p-values changed in two cases (vacuum extraction and BE).

### 3.1 IOL versus SOL at gestational age 41 + 0 – 41 + 6

In this group, in which pregnant women gave birth in week 41 of pregnancy, a comparison of the IOL (n = 23,772) and SOL groups (n = 62,611) showed that women in the IOL group were older (31.1 and 30.6 years, respectively, *P* < 0.001), with a higher body mass index (26 and 24.8 kg/m^2^, respectively, *P* < 0.001) and had a higher proportion of nulliparous women (57 % and 46.7 %, respectively, *P* < 0.001). In the IOL group, the proportion of women with a previous single caesarean delivery and tobacco user during pregnancy was lower than in the SOL group (1.9 % and 2.3 %, *P* < 0.001; 1.7 % and 2.0 %, *P* < 0.014, respectively) (Table 1).

**Table 1:** Patient demographic data presented as mean ± SD (standard deviation) or percentage.

	Week 41			Week 42	
	IOL n = 23,772	SOL n = 62,611	*A P-*value	SOL n = 4548	*B P-*value
**Age in years (n)**	31.1 ± 4.9 (23,772)	30.6 ± 4.6 (62,611)	<0.001	30.4 ± 4.7 (4548)	<0.001
**BMI (kg/m**^**2**^**)**	26.0 ± 5.1 (23,772)	24.8 ± 4.3 (62,611)	<0.001	24.0 ± 4.4 (4548)	<0.001
**Parity 0p % (n)** **1p % (n)** **> 1p % (n)**	57.0 (13,551) 27.1 (6437) 15.9 (3784)	46.7 (29,227) 35.4 (22,188) 17.9 (11,196)	<0.001	55.3 (2515) 28.8 (1309) 15.9 (724)	<0.001
**Previous single CS % (n)**	1.9 (463)	2.3 (1453)	<0.001	2.5 (112)	<0.001
**Tobacco user**	1.7 (773)	2.0 (1771)	0.014	2.8 (127)	0.004

A, IOL versus SOL in gestational week 41; B, IOL in gestational week 41 versus SOL in week 42; 0p, nullipareous; 1p, one earlier delivery; > 1p, more than one earlier delivery; CS, caesarean section.

Comparison of the IOL and SOL groups for primary maternal outcomes showed that various parameters were higher in the IOL group: caesarean sections (12 % and 4.6 %, *P* < 0.001), vacuum assisted delivery (8.7 % and 6.9 %, *P* < 0.001), and the proportion of women with blood loss of > 1000 ml (11 % and 8.3 %, *P* < 0.001). The rate of perineal rupture grade 3–4 did not differ significantly between the IOL and SOL groups. (Table 2).

**Table 2:** Foetal and maternal outcomes according to gestational age, presented as mean ± SD (standard deviation) or percentage, as indicated.

	Week 41			Week 42	
	IOL n = 23,772	SOL n = 62,611	A *P-*value	SOL n = 4548	*B P-*value
**Mode of Delivery**					
** N % (n)**	79.0 (18,785)	88.5 (56,389)	< 0.001	81.6 (3712)	< 0.001
** VE % (n)**	8.7 (2060)	6.9 (4333)	< 0.001	9.7 (450)	0.006
**CS % (n)**	12.3 (2927)	4.6 (2889)	< 0.001	8,5 (386)	< 0.001
**Blood loss >1000 ml % (n)**	11.0 (2607)	8.3 (5197)	< 0.001	11.4 (521)	0.148
**Perineal injury (grade 3 or 4) % (n)**	3.2 (756)	3.2 (1998)	0.459	3.6 (163)	0.080
**Apgar <4 at 5 min % (n)**	0.4 (105)	0.3 (164)	< 0.001	0.4 (16)	0.232
**Stillbirth % (n)**	0.18 (44)	0.07 (42)	< 0.001	0.16 (7)	0.478
**Meconium aspiration % (n)**	0.1 (28)	0.1 (74)	0.385	0.1 (4)	0.095
**pH (n)**	7.22 ± 0.07 (23,772)	7.23 ± 0.08 (62,611)	0.128	7.22 ± 0.07 (4548)	0.132
**BE mEq/l (n)**	−5.22 ± 3.0 (23,772)	−5.08 ± 4.4 (62,611)	0.157	−5.40 ± 3.1 (4548)	0.048
**Admission to NICU**	7.1 (1675)	7.2 (4546)	0.158	7.7 (350)	0.060
**Birth weight g(n)**	3804 ± 462	3083 ± 435	0.216	3889 ± 462	< 0.001

A, IOL and SOL in gestational week 41; B, IOL in gestational week 41 and SOL in week 42; BE, base excess; N, normal vaginal delivery; VE, vacuum extraction; CS, caesarean section; NICU, neonatal intensive care unit; n, number of patients¸ SD, standard deviation.

A logistic regression analysis after adjustment for confounding factors (women’s age, body mass index (BMI), parity, birth weight, tobacco use during pregnancy, and previous caesarean delivery) showed that the method of induction of labour (IOL) can influence the probability of delivery method by caesarean section (aOR 2.36, 95 %CI: 2.23–2.50, P < 0.001) and vacuum extraction (aOR 1.21, 95 %CI: 1.14–1.28, P = <0.001). The probability of blood loss of > 1000 ml also increased after IOL (aOR 1.22, 95 %CI: 1.16–1.29, P < 0.001). (Table 3).

**Table 3:** Odds ratio (OR) to quantify the strength of association between the method of labour commencement (IOL) and study parameters in two study groups: A) the IOL against SOL groups in gestational week 41, and B) the IOL group in gestational week 41 against the SOL group in week 42. Confounding variables are age, BMI, parity, birth weight, tobacco use, and previous caesarean delivery.

			A				B		
	Crude OR (95 % CI)		*P* value	Adjusted OR (95 % CI)	*P* value	Crude OR (95 % CI)	*P* value	Adjusted OR (95 % CI)	*P* value
**Vacuum extraction**	1.27 (1.20–1.34)		< 0.001	1.21 (1.014–1.28)	< 0.001	0.87 (0.78–0.96)	0.011	0.89 (0.79–1.00)	0.052
**Caesarean**	2.92 (2.77–3.08)		< 0.001	2.36 (2.23–2.50)	< 0.001	1.56 (1.39–1.74)	< 0.001	1.42 (1.27–1.60)	< 0.001
**Apgar <4**	1.75 (1.37–2.25)		< 0.001	1.54 (1.20–1.99)	< 0.001	1.25 (0.74–1.13)	0.393	1.17 (0.69–2.00)	0.543
**Admission to NICU**	0.97 (0.91–1.02)		0.309	.0.91(0.86 −0.97)	0.003	0.91(0.80 −1.02	0.126	0.87(0.77 −0.99)	0.038
**Stillbirth**	2.52 (1.65–3.85)		< 0.001	2.21 (1.43–3.41)	< 0.001	0.90 (0.37–2.16)	0.817	1.00 (0.44–2.24)	0.992
**Blood loss >1000 ml**	1.36 (1.29–1.43)		< 0.001	1.22 (1.16–1.29)	< 0.001	0.95 (0.86–1.05)	0.348	0.99 (0.89–1.09)	0.860
**Perineal injury (grade 3 or 4)**	0.99 (0.91–1.08)		0.901	0.85 (0.78–0.93)	< 0.001	0.88 (0.74–1.04)	0.147	0.90 (0.76–1.08)	0.286

CI, confidence interval; NICU, neonatal intensive care unit.

Comparison of the primary foetal outcome parameters between the IOL and SOL groups in gestational week 41 showed that the proportions of stillbirths in the IOL and SOL groups at delivery during gestational week 41 differed significantly (0.18 % and 0.07 %, respectively, P < 0.001), and the proportion was higher in the IOL group. (Table 2) This effect remained even after adjustment for potential confounders: maternal age, BMI, parity, newborn birthweight, tobacco use, and previous caesarean delivery (aOR, 1.21; 95 %CI: 1.43–3.41, P < 0.001). (Table 3) The proportion of newborns with apgar scores < 4 at five minutes was significantly higher in the IOL group than in the SOL group (0.4 % and 0.3 %, respectively, P < 0.001, Table 2), and the OR for apgar < 4 min at five minutes, even after adjustment for potential confounders, was higher after induced labour than after the spontaneous onset of labour (aOR, 1.54; 95 % CI, 1.20–1.99, P < 0.001). (Table 3).

There were no differences between the IOL and SOL groups in the proportion of neonates diagnosed with meconium aspiration and admission to NICU, or foetal blood pH values, BE, and birth weight. (Table 2).

### 3.2 IOL at gestational age 41 + 0–41 + 6 versus SOL at gestational age 42 + 0–42 + 6

We also investigated how the foetal-maternal outcomes differ between the pregnant women who gave birth after IOL in gestational week 41 and the women with expectant management and spontaneous onset of labour in gestational week 42. The number of patients in the IOL group was 16,832 and that in the SOL group was 4548. (Fig. 1).

Women in the IOL group were older (31.1 and 30.4 years, respectively, P < 0.001), with a higher body mass index (26 and 24 kg/m^2^, respectively, *P* < 0.001) and had a higher proportion of nulliparous women (57 % and 55.3 %, respectively, *P* < 0.001). In the IOL group, the proportion of women with a previous single caesarean delivery and tobacco user during pregnancy was lower than in the SOL group (1.9 % and 2.5 %, *P* < 0.001; 1.7 % and 2.8 %, *P* = 0.004, respectively). (Table 1).

Further comparisons of the maternal variables in the IOL and SOL groups revealed that the proportion of caesarean sections was higher (12.3 % and 8.5 %, *P* < 0.001), while the proportion of instrumental deliveries was lower (8.7 % and 9.7 % *P* = 0.006) in the IOL group. (Table 2) A logistic regression analysis, after adjustment for the aforementioned confounding factors, indicated that IOL may increase the probability of delivery by caesarean section (aOR 1.42, 95 %CI: 1.27–1.60, *P* < 0.001). However, the effect on delivery by vacuum extraction was likely influenced by factors other than just induction of labour (BMI, previous caesarean section, and weight of the child) (aOR 0.89, *P* = 0.052). (Table 3) This variable was affected by the sensitivity analysis, resulting in *P*-values becoming non-significant.

There was no significant difference between the IOL and SOL groups in the proportion of women with blood loss of > 1000 ml, perineal injury (grade 3 or 4), stillbirth, newborns with apgar scores < 4 at five minutes, diagnosis of meconium aspiration, newborns admitted to NICU, and pH value. However, the BE value differed between groups and was more negative in the SOL group (−5.40 vs −5.22, *P* = 0.048). This variable was also affected by the sensitivity analysis, which resulted in the *P*-values becoming significant. As expected, the newborns birth weight was significantly higher in the SOL group than in the IOL group (3804 and 3889 g, respectively, P < 0.001). (Table 2).

## 4 Discussion

In this registry-based study, a comparison of maternal and foetal outcome data using two methods of labour onset at different gestational weeks after the due date suggests that induction of labour at 41 weeks’ gestation may result in increased maternal and foetal morbidity and stillbirth risk compared to spontaneous onset of labour at week 41 of pregnancy. The risk of adverse effects on foetal outcome after IOL at week 41 was similar in the group of women who gave birth after SOL at week 42 of pregnancy.

An elective induction of labour generally reduces some risks associated with an ongoing pregnancy (e.g., the development of preeclampsia, oligohydramnios) [16]. All stillbirths beyond the estimated due date are always unexpected, because foetuses with recognized risk factors are usually delivered earlier. The most important independent risk factor for stillbirth is intra-uterine growth retardation (IUGR), which, according to an epidemiological study, can be found with stillbirth in about 50 % of cases at any gestational age [17]. In the present study, we excluded all pregnant women with known risk factors at enrolment, including those with newborns with a birth weight below the 10th percentile for gestational age at birth, so we consider that all the pregnant women in this study were in the low-risk group.

Prolonged pregnancies are associated with increased risks of foetal and neonatal mortality and morbidity [3], [13]. Interestingly, the risk of adverse effects on foetal and maternal outcomes (except for delivery methods) was similar in both the IOL group at week 41 and the SOL group at 42 weeks of pregnancy. However, both groups had a higher risk compared to the SOL group at week 41 of pregnancy. This can be interpreted as the risk of a negative effect on foetal and maternal outcomes increases over time after the due date. In a systematic review that quantified the risks of stillbirth and neonatal death according to gestational age after 37 weeks of gestation, the risk of stillbirth increased with gestational age [18]. However, the findings of earlier epidemiological studies are somewhat inconsistent [3], [19], [20]. Some earlier studies suggested that a policy of induction of labour at 41 weeks in prolonged pregnancy has some benefits, potentially improving the perinatal outcomes and reducing maternal complications [12], [13], whereas another study showed that after the induction of labour in post-date pregnancies, the rates of perinatal mortality and neonatal morbidity were similar to those in the expectant management group when the pregnancy was serially monitored in the antenatal period, although induction lowered the rate of caesarean section [21]. In contrast, another study suggested that induction prior to post-date in low-risk pregnancies is associated with few benefits and several adverse outcomes, and the final results did not support the routine use of induction before gestational week 41 + 0–41 + 6 [22]. Our results confirm that in low-risk prolonged pregnancies, the induction of labour in gestational week 41 can increase the risk of stillbirth compared with SOL in gestational week 41.

There are other risks associated with induced labour, including failed induction and an increased rate of caesarean section. Several earlier studies have suggested that the induction of labour is associated with an increased risk of emergency caesarean section, in both nulliparous and multiparous women, compared with the spontaneous onset of labour [23], [24]. A large multicentre trial conducted by Hannah et al. suggested that the induction of labour in prolonged pregnancy is associated with a reduction in the rate of caesarean section. However, the rates of perinatal mortality and neonatal morbidity were similar with the two management methods (expectant or induction) [21]. Subsequently, a Cochrane meta-analysis reported a significant reduction in perinatal mortality, without any increase in the risk of delivery by caesarean section, in a group of patients in whom labour was induced at 41 weeks of gestation compared with those in the expectant management group [25]. Our results show that the induction of labour in gestational weeks 41 increase the probability of deliveries with vacuum extraction and caesarean section compared to SOL in week 41 of pregnancy.

The risk of post-partum haemorrhage after IOL has been reported, with partly conflicting results, and some studies have suggested that IOL in low-risk singleton pregnancies is associated with greater blood loss [26], [27]. In contrast, several earlier studies were unable to demonstrate any difference in the volume of blood loss associated with labour after prolonged pregnancies in women with induced or spontaneous labour [13], [28]. The risk of blood loss of > 1000 ml at delivery was higher in the IOL group in gestational week 41 compared to the SOL group in week 41. Although analysis of our data suggested an influence of the method of delivery (IOL) on the risk of major blood loss at birth, in pregnant women who delivered after SOL at 42 weeks of gestation, both the proportion and the odds of blood loss > 1000 ml at birth were similar to those in the IOL group. As mentioned above, those patients also had a higher percentage of operative deliveries, which could have contributed to the larger volumes of blood loss.

A limitation of our study is that we were unable to present any data on the indications for induction or the methods used for labour induction, therefore, an effect of these factors on the results cannot be ruled out. Our results should also be interpreted with caution because this was a registry-based study with known limitations (e.g., missing information on data quality, unavailability of necessary information) and because differences in the routines of different maternal care centres can introduce heterogeneity into the data. In this study, the rate of missing data for some vaiables was substantial (around 30 %), so it can be mentioned as another limitation which can significantly impact the reliability and validity of our findings, especially when evaluating the condition of newborns. However, sensitivity analysis, as a crucial step in ensuring the robustness of our study’s findings, showed a marginal effect of missing data on the study’s results.

## 5 Conclusion

Our analysis of registry data, which is subject to the limitations, shows in prolonged pregnancy that both the commencement method of labour and gestational age can influence the health of the mother and child at birth. Analysis of previously recorded data shows that an earlier induction of labour in gestational week 41 may have a negative impact on foetal-maternal outcomes compared to SOL in the same week. The risk of adverse foetal-maternal outcomes may increase over time after the due date, and deliveries after SOL at 42 weeks of pregnancy may have the same risks as with IOL in gestational week 41. Further research is required, as our results should be verified with a large population-based prospective study.

## Author contributions

ST and LB were responsible for the study concept, design, and data collection. ST analysed the data. ST and LB interpreted the results. Both authors contributed to drafts of the manuscript, reviewed the results, revised the manuscript critically for important intellectual content, and approved the final version of the manuscript.

## CRediT authorship contribution statement

**Sahruh Turkmen:** Writing – review & editing, Writing – original draft, Visualization, Supervision, Project administration, Methodology, Investigation, Formal analysis, Data curation, Conceptualization. **Linnea Binfare:** Writing – original draft, Visualization, Software, Resources, Methodology, Data curation, Conceptualization.

## Declaration of Competing Interest

The authors have no conflicts of interest to declare in relation to this article.
